# Intermittent vs Continuous Administration of Nerve Growth Factor to Injured Medial Septal Cholinergic Neurons in Rat Basal Forebrain

**DOI:** 10.4236/nm.2014.52014

**Published:** 2014-04-30

**Authors:** Kenneth E. Miller, Gregory E. Frierdich, Robert H. Dillard, Robert H. Soriano, Dikla G. Roufa

**Affiliations:** Department of Anatomy and Cell Biology, Oklahoma State University Center for Health Sciences, Tulsa, USA

**Keywords:** Nerve Growth Factor, Medial Septal Nucleus, Choline Acetyltransferase, Alzheimer’s, Fimbria, Fornix

## Abstract

Many medial septal neurons of the basal forebrain are dependent on nerve growth factor (NGF) from the hippocampus for survival and maintenance of a cholinergic phenotype. When deprived of their source of NGF by axotomy, medial septal neuronal cell bodies atrophy and lose their cholinergic markers. This is similar to what is observed in the basal forebrain during Alzheimer’s disease (AD). In the present study, medial septal neurons were axotomized in female rats by way of a fimbria/fornix lesion. For fourteen days following axotomy, varying NGF doses (1 – 250 μg/ml) were administered to the lateral cerebral ventricle with either mini-osmotic infusion or daily injection. The responsiveness of medial septal neurons was evaluated with choline acetyltransferase immunohistochemistry. Within the mini-osmotic pumps, NGF activity diminished greatly during the first five days of implantation, but increased dramatically in the CSF after five days of infusion. The responsiveness of medial septal neurons to NGF was dose dependent and the ED_50_ for NGF injection was determined to be 14.08 μg/ml compared to 27.60 μg/ml for NGF infusion. Intermittent injections at varying intervals were evaluated with 30 μg/ml NGF over a fourteen-day time period (2, 3, 6, or 12 injections). No differences occurred in the number of choline acetyltransferase neurons from rats that received weekly injections to those that received daily injections of NGF. NGF administration has been suggested as a therapy for AD. The results of these studies continue to highlight the need for NGF stability within the delivery system and AD patient CSF, the choice of delivery system, frequency of administration, and the NGF dose for maintaining basal forebrain cholinergic neurons during AD.

## 1. Introduction

In the rat, medial septal/diagonal band (MS/DB) neurons of the basal forebrain establish connections with the hippocampal formation in late embryonic (E18–19) development [1] [2]. Basal forebrain cholinergic neurons develop from the ventral pallium [3] and are responsive to nerve growth factor (NGF) during development [4] [5]. NGF is synthesized for release by pyramidal neurons of the hippocampus and granule cells of the dentate gyrus [6] [7]. Once synaptic connections have been established, MS/DB neuronal terminals uptake and retrogradely transport NGF to neuronal cell bodies for survival and maintenance of cholinergic phenotype [8].

During Alzheimer’s disease (AD), plaques and tangles in the hippocampus disrupt the production, release, and uptake of NGF causing the atrophy and cell death of MS/DB cholinergic neurons [9]. Diminished acetylcholine release from MS/DB nerve terminals exacerbates the altered synaptic function of the hippocampal formation leading to a decline in consolidation of memories. Cholinesterase inhibitors, therefore, are a common therapy for AD [8] [9]. Exogenously supplied or gene-therapy delivered NGF has been proposed as alternative therapies for preservation of MS/DB neurons, maintenance of cholinergic function in the hippocampus, and attenuation of the progression of AD [10]–[12].

Similar to AD, medial septal (MS) neurons in rodents are deprived of their source of NGF following fimbria/fornix (FF) lesions causing MS neuronal atrophy and loss of cholinergic markers [13]-[15]. Exogenously supplied NGF rescues and maintains lesioned MS neurons and their cholinergic phenotype [16]-[18]. Many studies have used constant infusion or intermittent injection of NGF into the lateral ventricles after FF transection in rats [19] [20]. Dose response studies for infused NGF have been completed for MS neurons [21]-[24], but no direct comparison of the NGF dose responses for continuous infusion versus intermittent injections has been performed. In the current study, we evaluated the dose effect of NGF using daily intracerebroventicular (ICV) injections versus continuously infusion with mini-osmotic pumps for maintaining MS cholinergic neurons following a FF lesion. MS neurons were evaluated using immunohistochemistry for choline acetyltransferase (ChAT). After determining the NGF dose response for intermittent injections, we evaluated the frequency of NGF injections for maintaining injured MS neurons.

## 2. Materials and Methods

### 2.1. Animals

Female Sprague-Dawley rats (180 – 200 g) were used in this study. Animals were maintained on a 12 hr light-dark cycle and provided with continuous access to food and water. A total of 105 animals were used in this study. These studies were performed at the University of Oklahoma Health Sciences Center (OUHSC) and all procedures were approved by the OUHSC Institutional Animal Care and Use Committee (#90–135).

### 2.2. Fimbria/Fornix Lesion

All instruments and tubing were sterilized with steam or with 70% alcohol overnight. Animals were anesthetized with halothane. The head was shaved with an electric razor and placed in a stereotaxic unit. The scalp was swabbed with betadine and a midline incision was made to expose the skull. The overlying connective tissue was scraped and a drop of hydrogen peroxide was placed on the bone to enhance the appearance of bregma. The skull was dried with sterile gauze pads. A hole was drilled _−_1.0 mm from bregma and 1.0 lateral with a 1.80 mm diameter Trepan bur attached to a Dremel drill. The FF along with overlying cerebral cortex and corpus callosum was aspirated at a depth of 5 mm with a modified Pasteur pipette attached by rubber tubing to a side arm vacuum flask. Gel foam was placed in the skull hole.

### 2.3. Intracerebroventricular (ICV) Cannulation

Following FF lesioning, a small hole was drilled in the skull with a hand held twist drill at 1.5 mm lateral to bregma, rostral to the lesion hole. A cannula (see below) was inserted 4 mm through the hole into the lateral ventricle. A small hole was drilled into each parietal bone with a hand held twist drill and sterile stainless steel screws were screwed into the bone for anchoring dental acrylic. Activated dental acrylic was applied over the cannula and screws and allowed to dry. The scalp incision was closed with 9 mm wound clips and betadine was applied to the incision. The animals were removed from the stereotaxic unit, placed on a heating pad, and allowed to recover consciousness (5 – 10 minutes) after surgery. Afterwards, the animals were returned to their cages for two weeks.

### 2.4. Continuous Infusion with Mini-Osmotic Pumps

Mini-osmotic pumps (Alzet model 2002, flow rate 0.5 μl/hr, 14 day duration) were prepared the night before surgery. The pumps were filled with a dose of mouse NGF (5 – 250 μg/ml; D88 NGF-006, gift from Monsanto Co., St. Louis, MO) or cytochrome c (100 μg/ml; Sigma Chemical Company, St. Louis, MO) dissolved in artificial cerebrospinal fluid (ACSF) with 0.01% rat albumin. Solutions were filter sterilized using Costar Spin-X centrifuge filters (0.22 μm; Corning Life Sciences, Tewksbury, MA). Silicone rubber tubing (2 cm) was attached and the pumps were placed overnight in a beaker of sterile saline and 2 – 3 drops of betadine. After the cannula hole was drilled in the skull, the free end of the rubber tubing was attached to an L-shaped metal cannula (Small Parts, Inc., Miami, FL) and the cannula was inserted 4 mm into the lateral ventricle. The connective tissue under the skin of the back was spread with forceps and the pump was placed under the skin. Acrylic was applied over the skull and the incision sutured as described above. The pumps administered NGF or cytochrome c over a two week period (n = 5/group; n = 30 total).

### 2.5. Intermittent Injection

A 4 cm cannula was made from PE-20 tubing (BD Intramedic, Clay Adams, Parsippany, NJ). A collar of parafilm was made 4 mm from one end to prevent tubing from going too deep into the lateral ventricle. The other end was heat sealed with a soldering gun. The cannula was placed in 70% ethanol overnight. After a hole had been drilled in the skull, the cannula was placed into the lateral ventricle, acrylic applied, and the incision sutured. The sealed end of the cannula remained exposed through the incision for ICV injections over the two week period. For injections, the exposed end was cut and 5 μl of NGF solution (NGF in ACSF) was injected into the tubing with a 10 μl Hamilton syringe (Hamilton Company, Reno, NV). The syringe was removed and 5 μl of ACSF was injected with a separate Hamilton syringe to flush the cannula. The cut end was heat sealed with a soldering gun. This was repeated every other day throughout a two week period. When subsequent injections are made, the animals were restrained in a cloth towel. Injections with 1 – 90 μg/ml concentrations of NGF or 100 μg/ml of cytochrome c were delivered daily over a two week period (days 0 – 11; n = 5/group; n = 35 total).

After determining an NGF dose response for intermittent injection, another set of rats (n = 5/group; n = 25 total) was evaluated for the timing of intermittent injections of NGF. The NGF dose used for this study was 30 μg/ml, approximately 2× the ED_50_ concentration from the dose response curve. Animals received fimbria/fornix (FF) lesions and immediately were administered NGF into the lateral ventricle. NGF was administered into the lateral cerebral ventricle by intermittent injection with a 14 day survival. Four groups for the following injection regimens were formed: two injections—days 0, 7; three injections—days 0, 6, 12; six injections—days 0, 2, 4, 6, 8, 10, 12; twelve injections—days 0 – 11.

### 2.6. Immunohistochemistry

After two weeks of ICV administration of NGF or cytochrome c, the animals were anesthetized deeply with sodium pentobarbitol (60 mg/kg). The thorax was opened and the animal was transcardially perfused, first with 100 ml of saline followed by 400 ml of 2% paraformaldehyde, 2% picric acid in 0.1 M phosphate buffer, pH 7.2. The brain was removed and placed overnight in fixative (4_°C_). A coronal slab of brain tissue containing the basal forebrain was obtained with the use of a Precision Brain Slicer (Braintree Scientific, Braintree, MA). The brain tissue was affixed to a metal block with adhesive and placed into the water bath compartment of a Vibratome. The medial septum of the basal forebrain was sectioned at 60 μm, collected with a brush, and placed into a 6-well culture plate in phosphate buffered saline (PBS). Sections were washed in PBS three times for 10 min each, incubated in 10% phenylhydrazine (37_°C_) for 1hr, and washed in PBS for 30 min. Tissue sections were incubated in 10% normal rabbit serum for 1hr and placed for 48 hrs (4_°C_) in choline acetyltransferase (ChAT) antiserum (1:2000; Chemicon, Temeculca, CA). Antisera and normal sera were diluted in PBS with 0.3% Triton X-100. After 48 hrs, the tissue was washed in PBS and processed with avidin-biotin immunohistochemistry. The sections were placed on gelatin coated slides and dried. The sections were dehydrated in an ascending series of ethanols, cleared in xylenes, and coverslips were apposed with Permount.

### 2.7. Morphometry

All cell counting analyses were performed using Zeiss Axiophot microscope with a 10× objective. At this magnification, the medial septum from the lesioned side could be evaluated in 10 – 15 optical fields. Five sections from the medial septum (rostral to the anterior commissure) were used for quantification: one rostral, two middle, and two caudal sections. Neurons were counted with the aid of a Bioquant image analysis system (Nashville, TN). For each animal in every group, the total number of neurons in 5 sections was taken as a single value and these values were averaged for each group. These data were used to produce dose response curves and for calculation of ED_50_.

### 2.8. Data Analysis of MS ChAT Neurons

Groups were expressed as the mean ± SEM from individual animals. Statistical analysis included one-way ANOVA for group differences and Newman Keuls post-hoc test to assess individual group differences between control and lesioned animals.

### 2.9. PC12 Cells and NGF Evaluation

We also determined the amount of NGF activity in the cerebrospinal fluid (CSF) and mini-osmotic pumps from a group of non-injured rats that received continuous NGF infusion (n = 3/time point; n = 15 total). NGF (250 μg/ml) was infused into the lateral ventricle of unlesioned rats via mini-osmotic pumps (Alzet 2002, 0.5 μl/hr). The ACSF was removed from the mini-osmotic pumps and the CSF was removed from the cisterna magna of rats at 0, 1, 5, 10, or 14 days. Diluted ACSF or CSF fluid was applied to adrenal pheochromocytoma PC12 cells and the neurotrophic activity of the fluid was assayed by examining the length of neuritic extensions of PC12 cells.

PC12 cells were grown in T150 sterile flasks with filter tops (Corning) and used in bioassay between the PD and P28 passage. Cells were removed from their growth flasks by addition of 25 ml of a 0.05% trypsin, 0.53 mM EDTA solution (Gibco, Grand Island, NY) for 2 – 4 min and then manually dislodged from the bottom. PC12 cells were plated on diluted rat tail collagen (1:10 with sterile water) on 96-well Costar microtiter plates (Corning) at a density of approx. 15,000 cells/100pl media/well and grown for 24 hrs prior to sample addition. A standard curve for mouse NGF activity was generated by adding known concentrations of serially diluted mNGF to wells (10 ng/ml, 5 ng/ml down to 0.3 ng/ml) in triplicate. Neurite length was determined using a semi-quan-titative microscopic evaluation.

The amount of NGF-like activity present in experimental samples was determined by adding serial dilutions of the samples to wells and measuring the PC12 neuritic outgrowth relative to the mNGF standard curve. Serial dilutions were performed by adding 25 μl/well in a 96 well Half-Area plate (Costar) of PC12 media consisting of Dulbecco’s Minimal Essential Media, 10% fetal calf serum, 5% horse serum. Samples were added to the first well at 25 μl and serial dilutions were performed. The resulting materials were transferred to the wells containing the cells at 10 μl/well for 1:10 final dilution. Cultures were assayed after 2 – 4 days.

## 3. Results

### 3.1. NGF Activity in CSF and Mini-Osmotic Pumps

**Figure 1.**
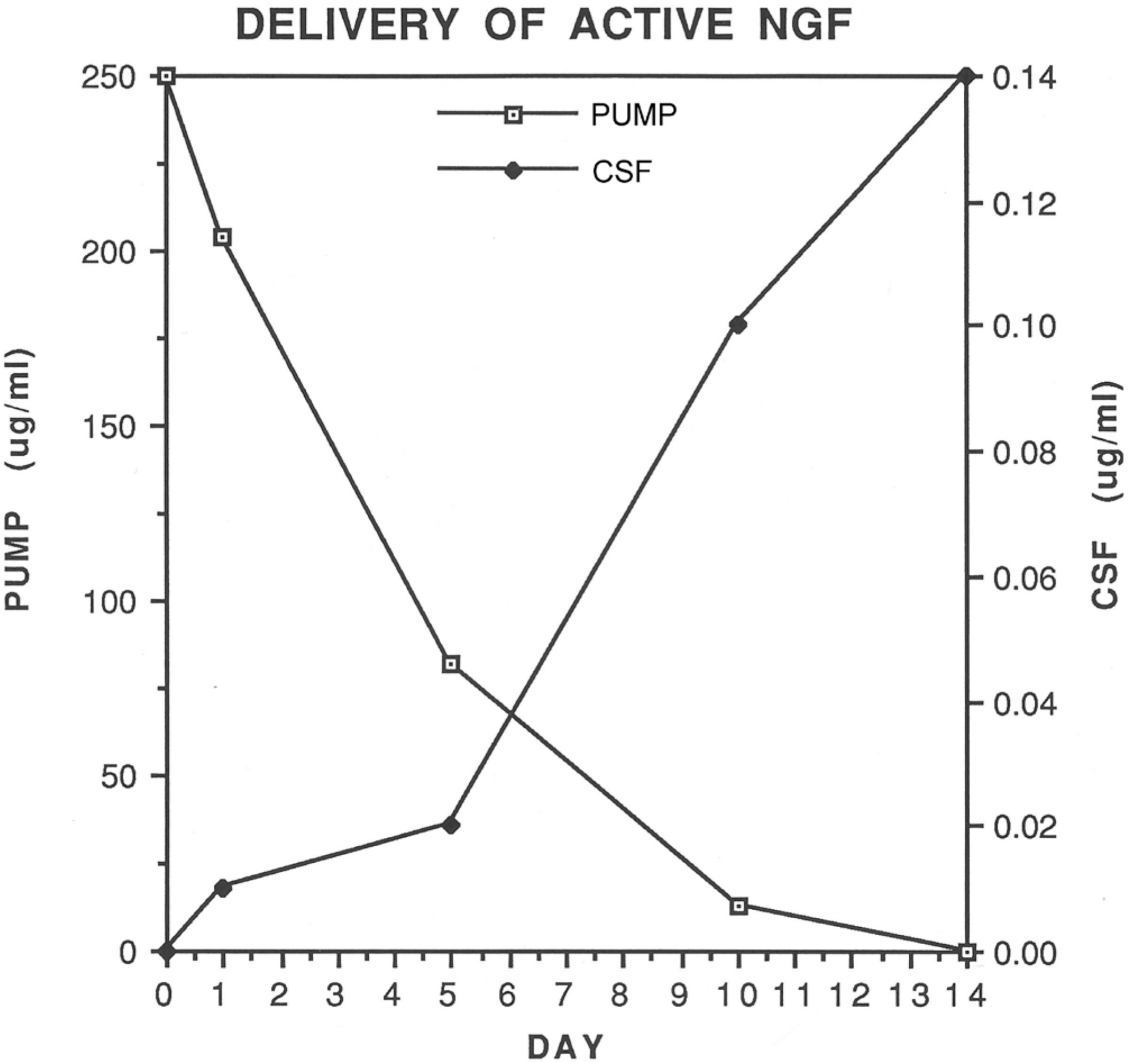
The NGF biological activity was evaluated both from mini-osmotic pumps and CSF of uninjured rats at 0, 1, 5, 10, and 14 days. The NGF activity (open squares) in the pumps was reduced by 70% at 5 days and was not detected by fourteen days. In comparison, the NGF activity in the CSF (closed squares) increased slowly over five days and rapidly increased from five to fourteen days.

By assessing neurite extension from PC12 cells, the NGF biological activity was evaluated from mini-osmotic pumps and CSF of uninjured rats at 0, 1, 5, 10, and 14 days (Figure 1). The NGF activity in the mini-osmotic pumps was reduced by 70% at 5 days and was negligible by fourteen days (Figure 1). The concentration of NGF activity in the CSF increased slowly over five days and then increased dramatically until fourteen days. A plateau for NGF biological activity was not reached during this time frame.

### 3.2. NGF Dose Response Following FF Lesions

**Figure 2.**
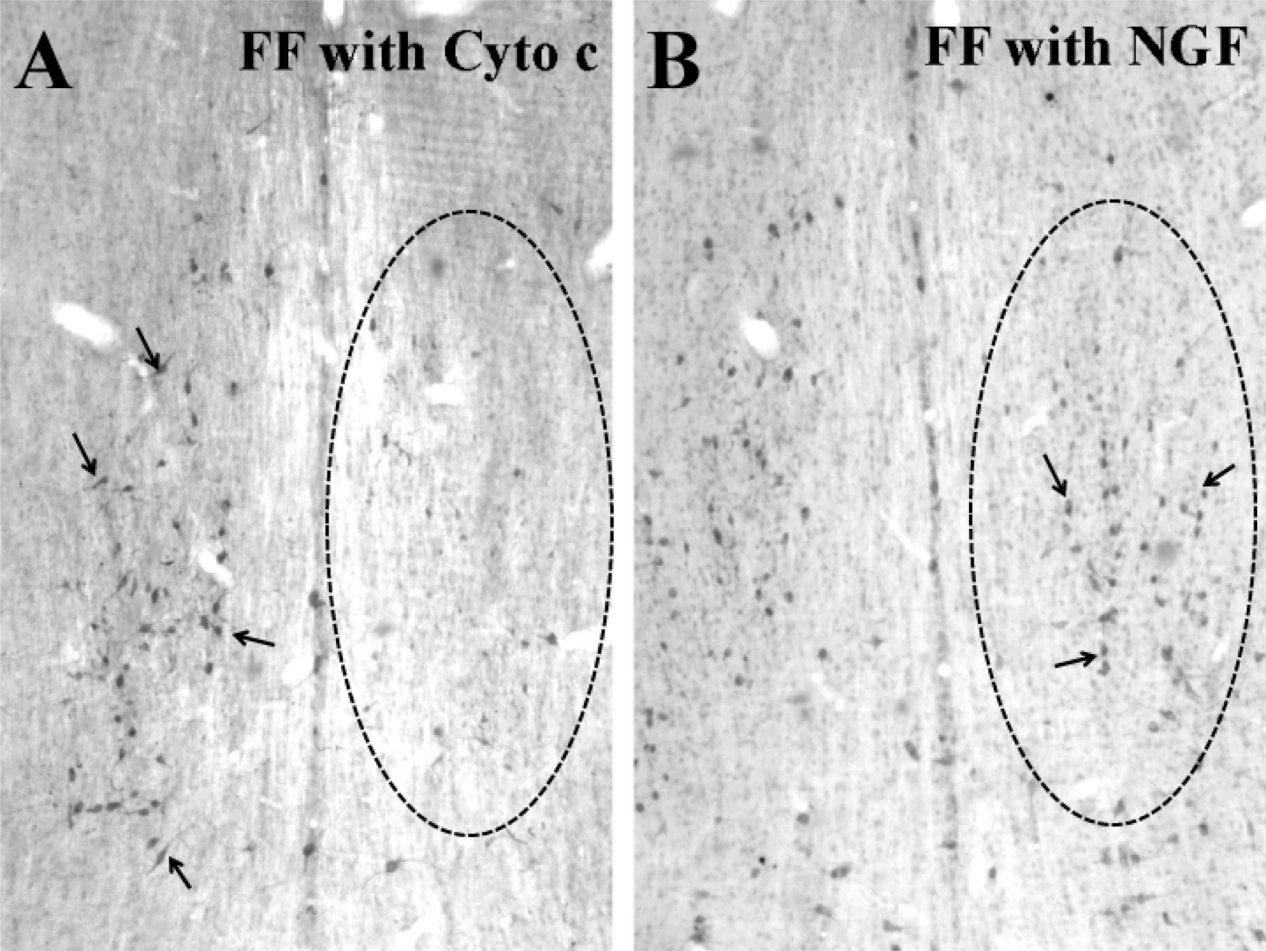
Choline acetyltransferase (ChAT) immunoreactive neurons (arrows) were identified in the medial septum with peroxidase immunohistochemistry. In A, ChAT immunoreactive neurons are largely absent (oval) in the medial septum fourteen days after fimbria-fornix (FF) lesion followed by cytochrome c administration to the lateral cerebral ventricle. In B, ChAT immunoreactive neurons (arrows) are retained (oval) in the medial septum fourteen days after fimbria/fornix (FF) lesion and continuous NGF infusion (125 μg/ml).

**Figure 3.**
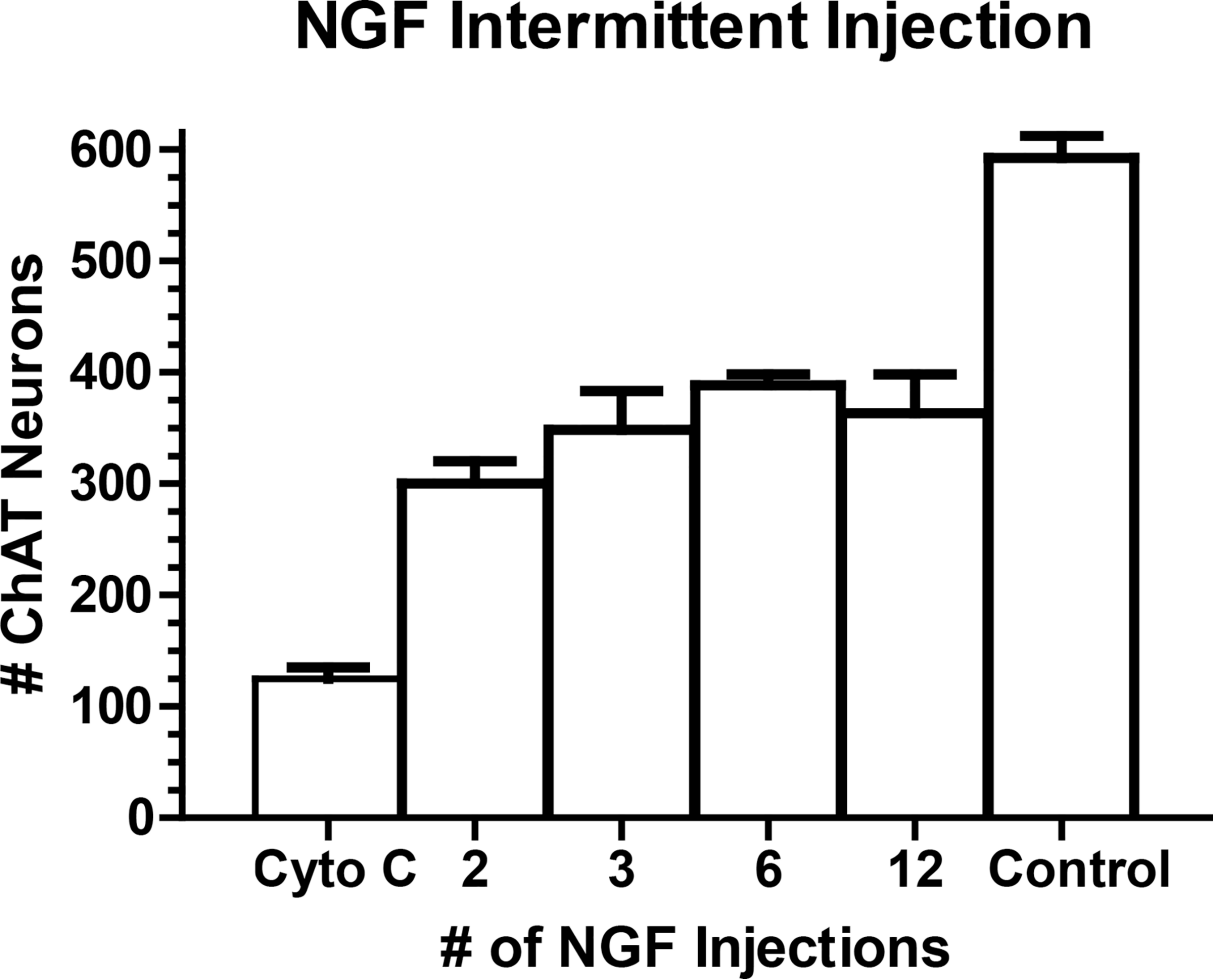
Rats with FF lesions were injected with 30 μg/ml of NGF at the time of axotomy. The total number of 30 μg/ml NGF injections varied over a 14 day time period: 2, 3, 6, or 12. ChAT immunoreactive neurons ranged between 300 – 400 cells for the various injection regimens. No significant differences were seen among the groups with FF lesions and varying intermittent NGF injections (Figure 3).

**Figure 4.**
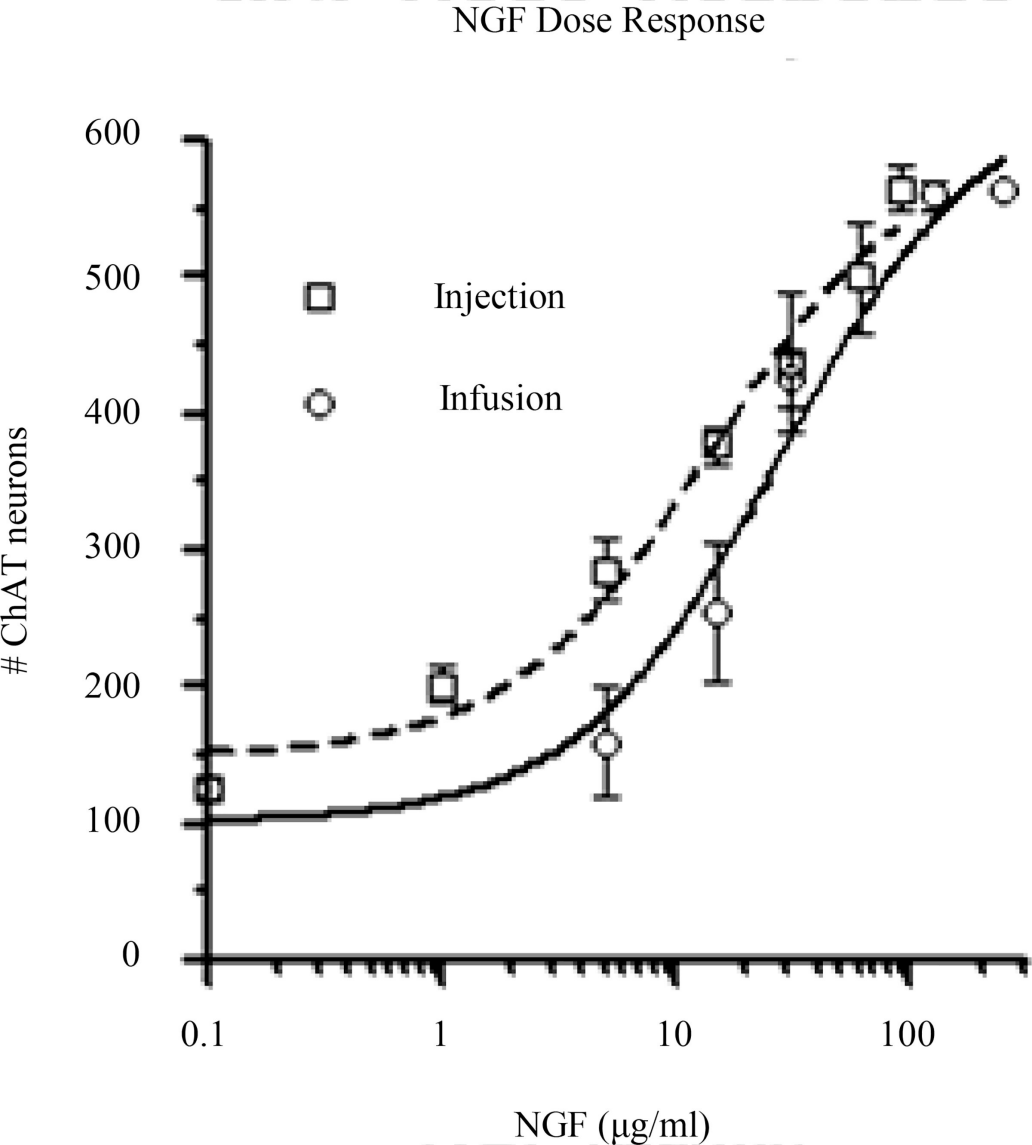
Animals received fimbria/fornix (FF) lesions and were administered NGF immediately. NGF was administered into the lateral cerebral ventricle by continuous infusion (open circles, full line) or intermittent injection (12 injections; open squares, dashed line) over a fourteen day period. ChAT immunoreactive neurons were totaled from five tissue sections of the MS. Unlesioned rats averaged approximately 600 neurons. An FF lesion with cytochrome c administration (control group) caused an 80% reduction of ChAT immunoreactive neurons compared to unlesioned rats. Administration of 100 μg/ml NGF by either intermittent injection or continuous infusion returned the number of ChAT neurons close to normal levels (>90%). MS neurons responded to NGF in a dose dependent manner (1 – 250 μg/ml), but there were differences between injection vs infusion application. The dose response curve for NGF injection was shifted to the left compared to the dose response curve for NGF infusion. The ED_50_ for NGF injection was 14.08 μg/ml and 27.60 μg/ml for NGF infusion.

In all cases, rats that received FF lesions were immediately administered NGF or cytochrome C into the lateral ventricle. NGF (1 – 250 μg/ml) was administered into the lateral cerebral ventricle by continuous infusion using mini-osmotic pumps or intermittent injection, 12 injections with 14 day duration. ChAT-immunoreactive (ChAT-ir) neurons (Figure 2) were counted in the MS and totaled from 5 sections (1 rostral, 2 middle, and 2 caudal sections). Unlesioned rats averaged approximately 600 neurons per five sections (Figure 3). An FF lesion with cytochrome C administration (control group) caused an 80% reduction of ChAT-ir neurons (125 neurons per five sections; Figure 3). Administration of >90 μg/ml NGF by either intermittent injection or continuous infusion returned the number of ChAT-ir neurons to near normal levels (90%; Figure 4). The responsiveness to NGF was dose dependent (1 – 250 μg/ml), but showed clear differences between injection vs infusion administrations. The dose response curve for NGF injection was shifted to the left compared to the dose response curve for NGF infusion. Furthermore, the ED_50_ for NGF injection was 14.08 μg/ml as opposed to 27.60 μg/ml for NGF infusion.

### 3.3. Timing of Intermittent NGF Injections Following FF Lesions

The timing of intermittent injections was studied by using a 30 μg/ml dose of NGF, approximately 2x the ED_50_ concentration from intermittent injection, dose response study. Animals received fimbria/fornix (FF) lesions and immediately were administered NGF into the lateral ventricle. A dose of 30 μg/ml NGF was administered into the lateral cerebral ventricle by intermittent injection (2, 3, 6, or 12 injections) with a 14 day survival. A total of 300 – 400 ChAT-ir neurons were detected in five MS sections from animals with this dosing regimen. No significant differences were seen among the groups with FF lesions and varying intermittent NGF injections (Figure 3).

## 4. Discussion

Nerve growth factor (NGF) is a neurotrophic factor produced by hippocampal and dentate neurons that maintains the cholinergic phenotype and/or survival of medial septal (MS) neurons. After fimbria/fornix (FF) lesioning in the rat, MS neurons atrophy and decrease the production of cholinergic markers, e.g., choline acetyltransferase (ChAT) [15]. Exogenous NGF promotes the phenotype and survival of injured cholinergic MS neurons [20] [22]. In order to further understand NGF’s neurotrophic effects on FF-lesioned MS neurons, the present study evaluated three topics: 1) the amount of NGF in the CSF infused by mini-osmotic pumps was evaluated at several time points during a fourteen day time period; 2) the dose response to NGF in injured MS neurons was compared using the infusion method versus a daily injection administration; 3) the response of injured MS neurons was evaluated with varying intervals of NGF injections over a fourteen day time period.

Intracerebroventricular (ICV) administration of NGF has been proposed for several decades as a treatment for Alzheimer’s disease (AD) and clinical trials with NGF delivered by ICV administration have been performed [25]-[27]. MS neurons atrophy and die during AD due to diminished synthesis, release, uptake, and transport of NGF. Exogenous NGF treatment during AD may maintain the survival of MS neurons, promote cholinergic neurotransmission in the hippocampal formation, and attenuate the progression of AD [26]. In AD patients, it will be important to maintain a constant therapeutic dose of NGF over long periods of time. Our results show that infusion of NGF into the CSF causes a significant rise in NGF levels over a two week period. With mini-osmotic pumps, there is a latency period of several days before substantial amounts of NGF are present in the CSF. This latency period could be overcome in AD patients with an initial bolus administration of NGF or with a scalable infusion system. Furthermore, the biological stability of NGF in an external device also is important to consider [28]. Our results from a fourteen day study indicate that NGF activity drops considerably over a five day period and continues to diminish over the remaining nine days. Stabilization of NGF or ease of refilling an external device will be of concern for delivering NGF with biological activity [28].

Comparison of two types of NGF administration can help to characterize the dose effectiveness of NGF for lesioned MS neurons [24]. In our study, daily injection of NGF was more effective than the mini-osmotic infusion. The injection regime yielded an ED_50_ for NGF of 14.08 μg/ml compared 27.60 μg/ml for infusion. The infusion ED_50_ differs from an earlier study (10 μg/ml) and may be due to the source of mouse NGF or the stability of the NGF in the mini-osmotic pump [22]. Comparison of the dose response curves for injection of NGF vs infusion indicated that efficacy was better with intermittent infusion (leftward curve and lower ED_50_). This may be due to the instability of NGF in the pumps or the amount of time that was needed to achieve optimal levels of NGF in the CSF. Nevertheless, pulsatile administration appears to be an effective mode of NGF delivery for injured MS neurons. With a lower ED_50_, intermittent injection may obviate negative NGF effects in AD patients [25].

Varying the timing between NGF injections did not alter the number of ChAT-ir neurons in the MS. It was surprising that 2 – 3 injections per fourteen days were as effective as daily NGF injections. In rats receiving 2 – 3 injections, NGF’s biological activity may have been maintained sufficiently in the CSF for several days or it may be that injured MS neurons do not require a continuous supply of NGF for maintaining their cholinergic phenotype. Although ChAT-immunoreactivity did not change with weekly versus daily NGF injections, it is not known if other aspects of cellular metabolism are altered by the weekly compared to daily injections.

## 5. Conclusion

The results from these studies allow us to make several conclusions. Administration of NGF to the CSF by either injection or infusion is dose dependent for maintaining axotomized cholinergic neurons. If NGF is to be used as a therapy for AD, then either route of administration could be used. Infusion of NGF into the CSF causes a significant rise in NGF levels over a two-week period. In AD patients, it may be possible to maintain a constant therapeutic dose of NGF over long periods of time. Injections of NGF seem to have long lasting effects on axotomized cholinergic neurons in that weekly injections of NGF were as effective as daily injections over a two-week period. AD patients may not need NGF application on continuous or a day-to-day basis, but weekly or biweekly administration may be sufficient.
